# The relationship between psychiatric morbidity and quality of life: interview study of Norwegian tsunami survivors 2 and 6 years post-disaster

**DOI:** 10.1186/s12888-016-0868-8

**Published:** 2016-05-31

**Authors:** Ajmal Hussain, Egil Nygaard, Johan Siqveland, Trond Heir

**Affiliations:** Division of Mental Health Services, Akershus University Hospital, 1478 Lørenskog, Norway; Department of Psychology, University of Oslo, P.O box 1094, Blindern, 0317 Oslo, Norway; Center for Child and Adolescent Mental Health, Eastern and Southern Norway (RBUP), P.O. box 4623, Nydalen, 0405 Oslo, Norway; Institute of Clinical of Medicine, University of Oslo, P.O box 1078, Blindern, 0316 Oslo, Norway; Norwegian Centre for Violence and Traumatic Stress Studies, University of Oslo, P.O box 181, Nydalen, 0409 Oslo, Norway; Groruddalen Community Mental Health Center, Outpatient psychiatric clinic, Division of Mental Health Services, Akershus University Hospital, P.O box 1000, 1478 Lørenskog, Norway

**Keywords:** Depression, Natural disaster, Posttraumatic stress, Quality of life, Tsunami

## Abstract

**Background:**

The study investigated the impact of psychiatric disorders on Quality of Life (QOL) cross-sectionally and longitudinally in a group of Norwegian tourists severely exposed to the 2004 tsunami.

**Methods:**

Sixty-two adult Norwegian tsunami survivors were interviewed face to face 2 years post-tsunami (T1) and 58 were interviewed again by telephone 6 years post-tsunami (T2). The majority (81 %) reported direct exposure to the waves, and 14 participants (23 %) lost a close family member in the tsunami. Psychiatric morbidity was measured by structured clinical interviews and QOL was assessed with WHO’s Quality of Life-Bref scale. Multiple linear regression analyses were performed to assess the independent effects of psychiatric disorders on QOL 2 and 6 years after the tsunami.

**Results:**

Psychiatric disorders, especially depression, but also PTSD and other anxiety disorders, were associated with reduced QOL. Psychiatric disorders were more strongly related to QOL at 6 years after the tsunami than at 2 years.

**Conclusions:**

Psychiatric disorders, and especially depression, is related to reduced QOL in a disaster exposed population. Post-disaster psychiatric disorders, such as PTSD and especially depression, should be addressed properly in the aftermath of disasters.

## Background

Research on health-related Quality of Life (QOL) has been well established over the past four decades, despite disagreements on the theoretical understanding of the concept of QOL [1]. The WHO defines QOL as individuals’ perceptions of their position in life in the context of the culture and value systems in which they live and in relation to their goals, expectations, standards and concerns [2]. The QOL research has mainly focused on QOL in somatic chronic conditions [3, 4], life-threatening diseases [5, 6], and severe psychiatric disorders, such as schizophrenia [7, 8], when investigating the effect of ill health on QOL. However, some studies have investigated the relationship between common psychological morbidity, i.e., anxiety and depression, and QOL [9–11], indicating that depressive and anxiety disorders may have a profound impact on health-related QOL that is independent of chronic somatic illness [12].

Major depressive disorder (MDD) is commonly associated with significant impairments in health-related QOL [13]. The Medical Outcomes Study found that persons with depression have comparable or lower QOL in regard to physical aspect, psychosocial aspect, and role functioning than those who have chronic medical conditions [14]. A meta-analytic review has also indicated poor QOL among anxiety disorder patients [15]. Further research has suggested that QOL impairments in anxiety disorders may be particularly prominent among patients with posttraumatic stress disorder (PTSD). This is not surprising given that PTSD results from serious traumatizing events, such as childhood abuse, rape, interpersonal violence, experience of war, captivity and torture [16, 17], which are likely to highly affect QOL.

However, the relationship between QOL and PTSD and other psychiatric disorders following a natural disaster has not been fully investigated and findings so far are somewhat mixed [18]. Some studies of posttraumatic stress and QOL among earthquake survivors report a relation between high levels of posttraumatic stress and poor QOL, with disaster exposure, financial loss and low levels of post-disaster support, accounting for the reduction in QOL [19–22]. In a longitudinal perspective, a negative impact of disaster on QOL was also reported among Hurricane Katrina evacuees 12 months after the storm [23], while there was no evidence for a long-term effect on QOL in a community sample eight years after an earthquake [24]. Another study found that there was no significant relation between exposure and health-related QOL in the aftermath of an oil spill disaster [25]. Few studies used structured diagnostic interviews or investigated impacts of psychiatric morbidity on QOL over time. The lack of longitudinal studies limits a cause-effect understanding of the relation between psychiatric disorders and QOL. None of the studies to date have investigated the relationship between psychiatric morbidity and different domains of QOL in a population that mostly escaped secondary disaster stressors.

In the present study, we investigated the impact of various psychiatric conditions on QOL cross-sectionally and longitudinally among Norwegian tourists severely exposed to the 2004 tsunami. In addition, we aimed to study whether there were any changes in the relationship between psychiatric morbidity and QOL from 2 to 6 years post-disaster.

## Method

### The event

Thailand was one of the countries affected by the 2004 Indian Ocean tsunami [26]. The province of Pang Nga was hit especially hard, with a devastating effect on the coastal community [27]. At the time of the tsunami, many Norwegians and other tourists were housed in Khao Lak in Pang Nga and experienced the horrors of the disaster [28]. Of the 84 Norwegians who perished in the disaster, 68 were housed in Khao Lak. The tsunami and its impact on tourists are described in greater detail in previous articles [29, 30].

### Study sample

Of the 80 Norwegian survivors above 18 years known to have been present in Khao Lak at the time of the tsunami, we were able to make contact with 75. Among them, 63 agreed to participate and were interviewed 2 years post-tsunami (T1). One participant did not respond to questions concerning quality of life at T1, and we could not locate four persons for the follow-up interviews 6 years post-tsunami (T2). Thus, the present study sample includes 62 participants at T1, 59 at T2 and 58 who participated in both assessments. There were no differences in age and gender between participants and those who did not participate at baseline [29]. The most common reason given for not participating was lack of time.

At the time of the first interview, none of the participants were involved in unsettled insurance cases related to the 2004 tsunami. At T1, 62.9 % were married or cohabitating, 69.4 % were employed, and 69.4 % had higher education (≥13 years). Only 33.9 % of the participants were the sole participants in their family, 54.8 % were one of two participants in their family, 4.8 % were one of three participants in their family, and 6.5 % were one of four participants in their family. Mean age at the time of the disaster was 40.6 years (*SD* = 11.3 years), and there were 33 women (46.8 %) and 29 men. There were no significant gender differences in age, employment or education.

Twelve participants (19.4 %) reported no direct exposure to the waves, 25 (40.3 %) were touched or chased by the waves, and 25 (40.3 %) were caught by the waves. Twenty-six participants (41.9 %) reported tsunami-related injuries. Of these participants, 13 were hospitalized. Fourteen participants (22.6 %) reported that a close family member died in the tsunami. There were no significant gender differences in disaster exposure, injuries or loss.

At T1, 37 % of the participants reported that they had sought professional help and received treatment for mental distress caused by the tsunami. Treatment mainly constituted psychotherapy alone or in combination with pharmacological treatment (mostly antidepressants).

### Measurements

We used the Structured Clinical Interview for DSM-IV Axis I Disorders (SCID-I) to assess tsunami related PTSD [31]. We made a full assessment of all the PTSD criteria for all participants. To assess other psychiatric disorders, we used the Mini International Neuropsychiatric Interview (MINI), which is a brief and valid structured diagnostic interview that covers 16 axis I disorders according to DSM-IV criteria [32, 33]. With some exceptions, MINI measures current psychiatric morbidity (symptoms experienced in the previous month). We used the Norwegian version of the MINI 5.0.0, which has been validated in the clinical setting and shown good psychometric properties [34]. We included modules for specific phobia and somatoform pain disorder from the MINI-plus [33].

QOL was measured with The World Health Organization Quality of Life-Bref scale (WHOQOL-Bref) [35, 36]. This is a self-report, abbreviated version of the WHOQOL-100. It contains 26 items divided into four domains and 2 general items. The first general item measures overall QOL, and the second general item measures overall satisfaction with own health. In the current study, we employ the terms perceived QOL and satisfaction with health, respectively, for these items. The four domains measure physical health (7 items), psychological health (6 items), social relationships (3 items), and environmental quality of life (8 items). The items are rated on a Likert scale from 1 to 5 to determine a raw item score. Domain scores are calculated by multiplying the mean of all items included within the domain by 4, giving domain scores ranging from 4 to 20. The transformation into scaled score is done to make the scores comparable with the WHOQOL-100. Domain scores are scaled in a positive direction (i.e., higher scores denote higher quality of life).

### Procedure

All of the Norwegian tsunami survivors who had been in Khao Lak at the time of the disaster and could be located were personally contacted by telephone and invited to participate in the study. An information letter was mailed prior to the call. At T1, the participants were interviewed face to face, mostly in their homes either by the first author alone (*n* = 49) or with a co-interviewer, specialist in clinical psychology (*n* = 13). The two interviewers were trained in structured diagnostic interviewing and experienced in the administration and use of other relevant psychometric instruments. Some of the exposure variables were coded based on information extracted from the interview. In these cases, the first codings were conducted in collaboration with the co-interviewer to reach consensus. At the follow-up (T2), all of the interviews were carried out by the first author as telephone-interviews, but the collaboration with the co-interviewer was maintained. Difficult diagnostic cases were discussed between the two interviewers to reach consensus. Analyzes after T1 showed that there were moderate to high correlations between the full scale score and the two general questions. Therefore, for practical reasons, QoL was measured only by the two general items of the WHOQOL-BREF in the follow-up. With some exceptions, the questionnaires were presented before conducting the diagnostic interviews.

### Statistics

Pearson Chi-square test was used to analyze grouped variables, Student’s t-test was used to analyze mean differences between groups, and Pearson correlation was used to analyze bivariate relation between two continuous variables. All continuous variables were approximately normally distributed, with both skewness and kurtosis between ±2; therefore, parametric tests were used.

Major depressive disorder and dysthymic disorder were grouped together as “depressive disorders”. All anxiety disorders except specific phobia and posttraumatic stress disorder (PTSD) were grouped together as “other anxiety disorders”. Alcohol and illegal drug dependency and misuse were grouped together as “substance disorders”.

Multiple linear regression analyses were performed to determine the independent effects of diagnoses on QOL 2 (T1) and 6 (T2) years post-tsunami. Four subdomains of QOL at T1 were used as dependent variables in four separate models. These subdomains were not measured at T2, and single questions concerning perceived QOL and health satisfaction were used as dependent variables at T2. The models predicting QOL and health satisfaction at T2 were rerun controlling for QOL and health satisfaction at T1. All multiple models controlled for age and gender. Confidence intervals for change in adjusted R-square were computed by the bootstrap BC_a_ procedure [37].

There was no missing information among the included participants on any of the analyzed variables. A significance level of .05 was used for all statistical tests. All statistical analyses were conducted using IBM SPSS Statistics, version 19.0, except bootstrap BC_a_ intervals for change in adjusted R-square, which used the R package boot [The R Foundation for Statistical Computing, Vienna, Austria].

## Results

The most prevalent current psychiatric disorder 2 years after the disaster was specific phobia (*n* = 18), followed by other anxiety disorders (*n* = 17), depressive disorders (*n* = 14), PTSD (*n* = 7) and substance disorders (*n* = 6). There was significant comorbidity between PTSD and both depression (*n* = 6, χ^2^ (62, 1) = 17.99, *P* < .001) and other anxiety disorders (*n* = 5, χ^2^ (62, 1) = 7.68, *P* = .006). None of the other diagnoses overlapped significantly. Women displayed simple phobias more often than men (45.5 % vs. 10.3 %, χ^2^ (62, 1) = 9.24, *P* = .002). There were no significant gender differences in depression, PTSD, other anxiety disorders or substance disorders.

### QOL 2 and 6 years post-disaster

The environment was the domain of QOL with the highest mean score 2 years after the tsunami, and physical health was the domain with the lowest scores (Table 1). There were no significant gender differences in any domains of QOL. All domains were significantly correlated (*r* from .43 to .69, all with *P* < .001). The single questions concerning perceived QOL and health satisfaction 2 years after the tsunami were highly correlated with all four domains of QOL (*r* from .57 to .69 for perceived QOL and *r* from .42 to .59 for health satisfaction, all with *P* < .001). Paired sample t-test indicated that there were no significant changes over time in perceived QOL or health satisfaction, as measured by a single question.

**Table 1:** Quality of life 2 years (T1) and 6 years (T2) after the tsunami

	T1 (*N* = 62)	T2 (*N* = 59)
	M (SD)	M (SD)
Physical health^a^	12.6 (1.9)	
Psychological health^a^	14.5 (2.2)	
Social relationships^a^	15.3 (3.1)	
Environment^a^	16.0 (2.7)	
Percieved quality of life^b^	4.0 (0.9)	4.1 (1.0)
Health satisfaction^b^	3.6 (1.2)	3.7 (1.1)

Table showing quality of life scores at T1 and T2. Mean scores (M) are given, measured with the World Health Organization Quality of Life-Bref scale (WHOQOL-Bref)

^a^Domain scores from WHOQOL-BREF (range 4-20); these were not measured 6 years post-tsunami

^b^Single question from WHOQOL-BREF (range 1-5)

### Association between morbidity and domains of QOL after 2 years

In the bivariate analyses, the four domains of QOL measured 2 years after the tsunami were significantly related to depression (Table 2). PTSD was significantly related to psychological health, social relationships, and environment, but not to physical health. Other anxiety disorders were significantly related to psychological health and social relationships, but not psychical health or environment. Specific phobias and substance disorders were not significantly related to any domains of QOL.

**Table 2:** Bivariate relations between psychiatric disorders and domains of quality of life 2 years post-tsunami (*N* = 62)

	Physical health	Psychological health	Social relationships	Environment
Depressive disorders	-2.84***	-3.17***	-2.95***	-3.37***
Specific phobia	0.03	-0.24	0.42	-1.01
PTSD	-1.37	-3.25***	-4.19***	-3.99***
Other anxiety disorders	-0.61	-1.34*	-2.43**	-1.13
Substance disorders	-0.29	-0.56	-1.97	-0.79

Figures are mean differences between those with and without psychiatric disorder as tested by Student t-tests. The direction of the difference is negative; thus, those without a psychiatric disorder had better quality of life than those with a psychiatric disorder

*P ≤ .05; **P ≤ .01; ***P ≤ .001

In the multiple regression analyses (Table 3), depression was the only psychiatric disorder that was significantly associated with the following three domains of QOL 2 years after the tsunami: physical health, psychological health, and environment. Only other anxiety disorders were significantly related to the social relationships domain. There were no other significant associations between psychiatric disorders and the four domains of QOL.

**Table 3:** Psychiatric disorders and their association with domains of quality of life 2 years post-tsunami (*N* = 62)

	Physical health	Psychological health	Social relationships	Environment
Depressive disorders	-3.26 (-4.42, -2.10)***	-2.62 (-3.93, -1.32)***	-1.69 (-3.63, 0.26)	-2.53 (-4.21, -0.84)**
Specific phobia	0.23 (-0.78, 1.24)	0.46 (-0.67, 1.59)	1.10 (-0.59, 2.79)	-0.48 (-1.95, 0.99)
PTSD	1.07 (-0.55, 2.69)	-1.02 (-2.84, 0.81)	-2.35 (-5.07, 0.37)	-1.92 (-4.28, 0.44)
Other anxiety disorders	-0.35 (-1.33, 0.64)	-0.67 (-1.78, 0.43)	-1.78 (-3.43, -0.13)*	-0.05 (-1.48, 1.38)
Substance disorders	-0.55 (-2.00, 0.89)	-0.89 (-2.51, 0.74)	-1.80 (-4.23, 0.63)	-0.89 (-2.99, 1.22)
Adjusted R^2^	0.33	0.36	0.28	0.26

Multiple linear regression analyses showing various psychiatric disorders and their association with four domains of quality of life 2 years post-tsunami. Figures are unstandardized regression coefficients, with 95 % confidence intervals in parenthesis. All independent variables were entered simultaneously into the models. Age and gender were controlled in all models. Neither age nor gender was a significant predictor of quality of life in any of the models. Quality of life is measured by four domains from WHOQOL-BREF

*P ≤ .05; **P ≤ .01; ***P ≤ .001

### Association between morbidity and QOL after 2 and 6 years

The multiple regression analyses showed that depression was related to perceived QOL 2 and 6 years after the tsunami (Table 4). PTSD and specific phobias were significantly related to perceived QOL after 6 years, but not after 2 years. Both depression and PTSD, but not social phobias, remained significantly associated with perceived QOL after 6 years when controlling for perceived QOL after 2 years.

**Table 4:** Psychiatric disorders at 2 years and their association with quality of life 2 and 6 years post-tsunami (*N* = 58)

	Perceived quality of life at T1	Perceived quality of life at T2	Perceived quality of life at T2 controlling for T1
Depressive disorders	-1.35 (-1.84, -0.86)***	-1.43 (-1.92, -0.95)***	-0.85 (-1.41, -0.29)**
Specific phobia	-0.11 (-0.52, 0.30)	-0.40 (-0.80, 0.01)*	-0.35 (-0.72, 0.02)
PTSD	-0.44 (-1.16, 0.28)	-0.85 (-1.55, -0.14)*	-0.66 (-1.31, -0.01)*
Other anxiety disorders	-0.04 (-0.46, 0.69)	-0.13 (-0.55, 0.29)	-0.12 (-0.49, 0.26)
Substance disorders	-0.08 (-0.73, 0.57)	-0.14 (-0.78, 0.50)	-0.11 (-0.69, 0.48)
Perceived quality of life at T1			-0.43 (-0.69, -0.18)***
Adjusted R^2^	0.51	0.62	0.69

Multiple linear regression analyses of psychiatric disorders at 2 years association with perceived quality of life 2 (T1) and 6 years (T2) post-tsunami. Figures are unstandardized regression coefficients, with 95 % confidence intervals in parenthesis. All independent variables were entered simultaneously into the models. Age and gender were controlled in all models. Gender and age were not significant predictors of perceived quality of life in any of the models. Perceived quality of life is here measured with a single question from WHOQOL-BREF: “How would you rate your quality of life?”

* *P* ≤ .05; ** *P* ≤ .01; ***P ≤ .001

Depression was related to health satisfaction at both time points and remained significant after 6 years when controlling for health satisfaction after 2 years. None of the other psychiatric disorders were significantly related to health satisfaction at any time point in the multiple regression analyses.

Explained variance after 2 years in the regression models in Tables 4 and 5 were 51 and 22 % for perceived QOL and health satisfaction, respectively, and increased to 62 and 52 %, respectively, 6 years post-disaster. The increase in explained variance in perceived QOL was not significant. However, the increase in explained variance in health satisfaction was significant (95 % CI of ∆adjusted *R*^*2*^ = 0.05 to 0.56).

**Table 5:** Psychiatric disorders at 2 years and their association with satisfaction with health 2 and 6 years post-tsunami (*N* = 58)

	Health satisfaction at T1	Health satisfaction at T2	Health satisfaction at T2 controlling for T1
Depressive disorders	-1.13 (-1.94, -0.31)**	-1.89 (-2.48, -1.31)***	-1.51 (-2.08, -0.95)***
Specific phobia	-0.35 (-1.02, 0.33)	-0.24 (-0.73, 0.25)	-0.12 (-0.56, 0.32)
PTSD	-0.12 (-1.30, 1.07)	0.30 (-0.55, 1.15)	0.34 (-0.42, 1.10)
Other anxiety disorders	-0.16 (-0.86, 0.54)	-0.26 (-0.76, 0.24)	-0.20 (-0.65, 0.25)
Substance disorders	0.33 (-0.75, 1.41)	-0.04 (-0.81, 0.73)	-0.15 (-0.84, 0.54)
Health satisfaction at T1			-0.34 (-0.52, -0.15)***
Adjusted R^2^	0.22	0.52	0.62

Multiple linear regression analyses of psychiatric disorders at 2 years association with satisfaction with health 2 (T1) and 6 years (T2) post-tsunami. Figures are unstandardized regression coefficients, with 95 % confidence intervals in parenthesis. All independent variables were entered simultaneously into the models. Age and gender were controlled in all models. Gender was not a significant predictor of Health satisfaction in any of the models. Age was significantly related to Health satisfaction at T1 (*B* = -0.03, 95 % *CI* = -0.05 to 0.00, *p* = .05), but was not significant in any of the other models. Health satisfaction is here measured with a single question from WHOQOL-BREF: “How satisfied are you with your health?”

*P ≤ .05; **P ≤ .01; ***USP ≤ .001

A significant number of persons with depression had lost a close family member in the tsunami (8 of 14, 57.1 %) compared to those without a diagnosis of depression (6/48 persons, 12.5 %) (χ2 (1) = 12.4, *P* < .001). We ran all of the multivariate analyses again while controlling for loss. We did not find any changes in results for depression in the multiple regression models.

## Discussion

### Discussion

We found that depression, PTSD and other anxiety disorders were associated with reduced QOL among Norwegian tsunami survivors. This is consistent with other studies of natural disaster survivors [22, 23]. In particular, depression seems to exert a broad negative effect on QOL, including overall measures of perceived QOL and health satisfaction. This finding is in agreement with many epidemiological studies [38, 39] and clinical studies [40]. However, there is no clear evidence in the literature that depression consistently has a more pronounced negative effect on QOL compared to other psychiatric disorders. It appears as though the disorder severity and its longitudinal course determine the associated reduction in QOL rather than a particular psychiatric condition per se [41]. In addition, due to the significant comorbidity between depression and PTSD in the current study, the role of PTSD may have been underestimated.

PTSD seems to have a significant negative effect on certain aspects of QOL, including psychological health, social relationships, and environment. The negative effect of PTSD or posttraumatic stress on elements of environment-related QOL is documented in previous disaster studies [19, 42]. Still, we found these effects rather surprising, as Norwegian tourists were repatriated within a short time to stable home communities [43, 44]. Environment-related QOL includes items such as financial resources, physical safety and security, health and social care, home environment, new opportunities, opportunity for recreation, physical environment, and transportation, which are likely to be negatively affected in the aftermath of a large-scale disaster. Thus, as the majority of our population escaped most of these secondary disaster stressors, one would expect a reduced impact of disaster exposure and subsequent PTSD symptoms on environment-related QOL.

The strong negative relationship between psychiatric disorders and psychological health- and social relationship-related QOL was expected, as poor mental health is likely to affect these domains [45]. The adjusted model showed that depression exerted the main negative effect on psychological health, whereas other anxiety disorders had the most significant effect on social relationships. One possible explanation for this finding might be that social relations were strengthened within the families after the tsunami, especially in depressed people. A previous study of Norwegian parents who experienced the 2004 tsunami reported an increase in family care and appreciation for family values [46]. These changes remained stable 2 years after the tsunami and are likely to have occurred in our population, as many of the participants lived within a family constellation with children. Such strengthened family cohesion may have buffered the likely negative effect of depression on social relationships.

Of the psychiatric disorders, physical health-related QOL was only related to depression and remained significantly related to depression after adjustment for other psychiatric disorders. One explanation could be that the measure of physical health contains items that overlap with depression, i.e., activities in daily living, energy and fatigue, sleep, pain, and work capacity. Of note, pre-disaster physical health was generally good in the current study population and few suffered severe injury [29], which reduces the possibility of overlap between a severe somatic condition and depressive symptoms.

The prevalence of specific phobias was relatively high, but this condition was not significantly related to QOL. We have previously shown that specific phobias had little negative impact on socio-occupational functioning among Khao Lak survivors compared to depression and PTSD [29]. The psychopathological effect of specific phobias in disaster populations seems to be relatively mild, as survivors can avoid phobic exposure related to specific situations without having any major impact on their daily life activities.

We did not find a negative effect of substance disorders on QOL. Although there has been less focus on QOL in the addiction field, available evidence suggests that QOL is generally poor among active substance abusers and treatment seekers [47, 48]. However, the majority of those who were diagnosed within this cluster/group in the present study did not have a severe form of substance abuse or dependence. In addition, the use of MINI may lead to an over-diagnosis of alcohol and substance misuse in non-clinical settings.

We used the two general items from WHOQOL-BREF to assess any changes in QOL over time. We found no change in the overall score for perceived QOL and health satisfaction from 2 years to 6 years after the tsunami. However, we found that psychiatric morbidity showed an overall increase in explained variance for both perceived QOL (51 to 62 %) and health satisfaction (22 to 52 %) from 2 to 6 years. All of the predictors were measured 2 years after the tsunami and more than 3 years before the last measure of QOL. Thus, we expected a decrease in the relation between psychiatric morbidity and QOL over time, as posttraumatic stress and other psychopathology tend to decrease with time after disasters [49, 50]. In addition, a correlation tends to weaken as the time between the measurements increases. One interpretation of the present finding may be that individuals who recover experience an increase in QOL, whereas individuals who do not recover experience either an increase in symptom level and subsequent poorer QOL or merely a negative change in perception of QOL with time [51, 52]. We cannot rule out that other variables, i.e. changes in socioeconomic status and aging process, may have contributed to strengthen the relationship between psychiatric morbidity and QOL over time. Another finding that supports the idea that changes occurred from 2 to 6 years post-disaster is that both PTSD and depression showed a reinforced negative relation to perceived QOL from T1 to T2. A similar change also occurred between depression and the construct of health satisfaction in QOL.

We believe that development of chronic mental illness may lead to a worsening of QOL due to deterioration of social relations, inappropriate use of own personal resources, apostasy from work, and increasing social isolation [52]. A study of earthquake survivors showed that QOL was worsened with time among those who received less relief support in the aftermath of disaster [53]. This may be opposed to chronic somatic conditions, for which many persons successfully adapt to symptoms, maintain social relations and are able to focus on their personal resources [54]. However, some studies also indicate that long-lasting depression may have debilitating effects on work and psychosocial functioning even after significant reduction in depressive symptoms [39, 54, 55]. As work and psychosocial functioning are closely linked to QOL, we cannot rule out the occurrence of the latter among some of the persons in the population.

### Methodological considerations

There are some limitations in the study. The small sample size yields low power for many of the analyzes. This suggests that caution must be made regarding the conclusions. In addition, the relatively small sample size limited detailed analyses i.e. of all the individual psychiatric disorders and other subgroups, as the results would have been more uncertain and difficult to interpret. Although we achieved a high response rate, there is still possibility of selection bias, as the main reason for not participating was lack of time, meaning that non-participants had a busy life, which may indicate a good quality of life and a good mental health. However, it is unclear how this assumption may have affected our results. The majority of the sample had at least one other family member experience the trauma with them which may have facilitated a feeling of strengthened social support and thereby influenced the results.

Telephone interviews were conducted in the 6-year follow-up compared to face-to-face interviews in the 2-year study, which could have affected the results. However, telephone interviews are often used in epidemiological research and are a valid method of assessing structured information such as axis I psychiatric disorders [56].

Generally, the present findings may be limited to populations that experience traumatic events with a sudden impact and relatively brief exposure and may not be applicable to other populations that are exposed to chronic stressors. One may also argue that this sample of Norwegians, having the possibility to spend their Christmas holiday abroad, may represent a more privileged sub-population of the Norwegian population. It is previously described that The Norwegian tourist population that experienced the tsunami did not differ from the general Norwegian population with regard to employment and marital status [57], though they had a higher than average education level and family constellation with children, which may indicate higher than average levels of socioeconomic status.

It is suggested that the prevalence of psychopathology documented in studies after natural disasters is generally lower than that documented after man-made disasters [50]. However, in studies of natural disasters it is more difficult to explicitly identify groups of persons who can be considered direct victims. Natural disasters often affect large areas and the study samples predominantly include persons from a broader area affected by the disaster. When studying persons who were in the more heavily affected areas at time of the disaster, like our population, higher prevalence’s of psychopathology are reported [58, 59].

The measure of QOL at T2 was restricted to perceived quality of life and health satisfaction measured with two single questions. This limits a thorough understanding of changes observed in the relation between psychiatric morbidity and QOL.

There are several strengths in the study. We achieved a relatively high response rate at T1 and the attrition rate between T1 and T2 was low, reducing the risk for bias. We used structured clinical interviews to detect a broad range of psychiatric conditions rather than covering a few disorders through self-reported questionnaires. We asked additional questions for the most relevant psychiatric disorders in order to follow the trajectories of these conditions. The study population was rather homogeneous due to the nature of the travel regarding work, family, finance, and physical and mental health. The relatively fast evacuation of the Norwegian tourists reduced the impact of secondary disaster stressors, limiting the impact of trauma to the primary tsunami experience. Finally, regardless of impairment level, the present sample received affordable and easily accessible medical and psychiatric care as well as ample community support [60, 61].

## Conclusions

In the current study of Norwegian tsunami survivors, we found that both depression and PTSD were negatively correlated with QOL both cross-sectionally and longitudinally. Psychiatric disorders, especially depression, must be addressed properly in the aftermath of disasters, as they have a continuously negative effect on QOL.

## Abbreviations

CI, Confidence interval; DSM-IV, Diagnostic statistical manual of mental disorders, fourth edition; M, Mean; MDD, Major depressive disorder; MINI, Mini international neuropsychiatric interview; PTSD, Post-traumatic stress disorder; QOL, Quality of life; SCID-I, Structured clinical interview for DSM-IV, axis I disorders; SD, Standard deviation; WHO, World Health Organization; WHOQOL-Bref, The World Health Organization Quality of Life-Bref scale
